# Influence of human chorionic gonadotropin (hCG) on *in vitro *growth of *Plasmodium falciparum*

**DOI:** 10.1186/1475-2875-8-101

**Published:** 2009-05-14

**Authors:** Clinton K Pong, Audrey Davidson Thévenon, James Ainong Zhou, Diane Wallace Taylor

**Affiliations:** 1 Department of Tropical Medicine, Medical Microbiology and Pharmacology, John A Burns School of Medicine, University of Hawaii, 651 Ilalo Street, Honolulu, HI, 96813, USA; 2 Department of Biology, Georgetown University, 37th and O Streets, NW, Washington DC, 20057, USA; 3 AZ DataClinic, Inc, Rockville, MD, 20850, USA

## Abstract

**Background:**

During pregnancy, women are more susceptible to *Plasmodium falciparum *infections and frequently have a higher parasitaemia than non-pregnant women. Several mechanisms are responsible for their increased susceptibility, including down-modulation of immune responses that aid in parasite clearance and sequestration of infected erythrocytes in the placenta. Early in pregnancy, a third mechanism may contribute to higher parasitaemia, since it has been reported that addition of human chorionic gonadotropin (hCG) to *in vitro *cultures of the NF54-strain of *P. falciparum *results in increased parasite growth rates. The goal of this study was to further examine the effect of hCG on *P. falciparum *growth.

**Methods:**

The NF54-3D7, FVO and 7G8 strains of *P. falciparum *were cultured *in vitro *with various physiological concentrations of hCG purchased from three sources. Infected erythrocytes were also co-cultured with a human cell line that naturally secretes hCG.

**Results:**

Results from 14 experiments using different combinations of parasite strains and concentrations of hCG from different sources, as well as the co-culture studies, failed to provide convincing evidence that hCG enhances parasite growth *in vitro*.

**Conclusion:**

Based on these data, it seems unlikely that hCG has a direct effect on the rate of parasite growth early in pregnancy.

## Background

Women, especially primigravidae, are more susceptible to the harmful effects of *Plasmodium falciparum *infections during pregnancy than non-pregnant women [1,2]. They are more likely to be slide-positive for malaria, have a higher parasitaemia, and develop anaemia. As a result, pregnant women are at an increased risk of clinical illness and poor pregnancy outcomes. A combination of factors contributes to higher parasitaemia and severity of disease. Physiological and immunological changes that occur during pregnancy alter immune responses that aid in parasite killing [3-5] and infected erythrocytes (IE) reach high numbers in the intervillous space (IVS) of the placenta due to sequestration [6]. A third possible reason for high parasitaemia was suggested by Rohrig *et al*, who found that the pregnancy-associated hormone, human chorionic gonadotropin (hCG) increased the growth rate of *P. falciparum in vitro *[7]. This finding has lead to the speculation that *P. falciparum *parasites may grow at a faster rate *in vivo *during the early part of pregnancy. A large number of studies have confirmed the importance of the first two mechanisms, but studies confirming the influence of hCG have not been reported.

A role for hCG in enhancing parasitaemia during pregnancy seems plausible. It is produced by syncytiotrophoblasts, the cell type that lines the IVS where high parasitaemias are found. hCG is released into the blood starting early following conception, reaches peak concentrations between 8 to 12 weeks of gestation, and then decreases to low levels by the early part of the second trimester [8]. The peak of hCG immediately precedes the period when women are reported to be the most susceptible to malaria. In surveying the literature, Brabin found that in many studies the period of highest prevalence of malaria and parasitaemia was during the 13–16 weeks of pregnancy [1]. Recently, a large-scale study found that hCG levels are slightly higher in primigravidae, the population of women most susceptible to malaria, and that hCG levels decrease with gravidity [9]. Thus, current data are consistent with the possibility that hCG enhances parasitaemia during pregnancy.

The purpose of this study was to further evaluate the effect of hCG on *P. falciparum *growth *in vitro*. In the sentinel study, hCG was purchased from Ferring (Germany) and added to cultures of the NF54 strain of *P. falciparum*. Unfortunately, hCG from this source was no longer available when we conducted the study. Previous studies have shown that significant differences in the composition and levels of contaminating bioactive molecules exist among commercial preparations of hCG [10,11]. For example, some of the properties initially ascribed to hCG were later found to be due to contaminating Epithelial Growth Factor [11] and serine proteases [12]. Therefore, in the current study purified hCG from three suppliers were used and their composition was compared by SDS-PAGE. In addition, IE were co-cultured directly with a human cell line (BeWo) that secretes hCG [13]. To determine if hCG enhanced the growth rate of IE in general, three strains of *P. falciparum *were employed, including NF54-3D7, FVO originally isolated from south-east Asia that has been maintained in long-term culture, and the 7G8 strains which is a fast-growing, chloroquine-resistant strain. Results from the *in vitro *studies using various combinations of parasite strains and sources of purified hCG (n = 14 experiments) failed to provide convincing evidence that hCG enhances the growth rate of *P. falciparum in vitro*.

## Methods

### Sources of hCG

HCG was purchased from the following companies. Sigma: Chorionic gonadotropin human, product number CG5, that contains approximately 5,000 IU/vial (Sigma, St. Louis, MO); Calbiochem, hCG: purified from human urine, standard grade, catalog number 230734, which contained ~3,000 IU hCG/ml based on the WHO standard IRP75/551 (Calbiochem.com); and Cell Sciences, Inc.: ultra pure, catalog number CRC101B, purified by a proprietary chromatographic technique containing 3,559 and 5,000 IU/ml based on IRP reference 75/551 (Cell Sciences, Inc., Canton, MA). Lyophilized hCG was reconstituted in ddH20 as recommended by the manufacturers and then diluted in complete culture medium (see below).

### In vitro culture of *P. falciparum*

*Plasmodium falciparum *of the NF54-3D7, FVO and 7G8 strains were maintained in continuous *in vitro *cultures based on the method of Trager and Jensen [14]. Parasites were cultured in A+ RBC at a 5% haematocrit in RPMI-1640 supplemented with 4.5g/L of D-glucose, 2.383 g/L of HEPES, 0.02 mg/mL of hypoxanthine, 1.5g/L of sodium bicarbonate, 0.11 g/L of sodium pyruvate, and 0.5% Albumax II (Gibco, Invitrogen). Parasites were grown in 96-well microtiter plates at 37°C in the presence of 5% CO_2_, 5% O_2 _and 90% N_2_.

Fourteen *in vitro *experiments were conducted using the 3D7 and FVO strains of *P. falciparum*. Triplicate microtiter wells were seeded with ~0.5% parasitaemia in the absence of hCG (control) or with two-fold serial concentrations of hCG ranging from 12.5 to 200 IU hCG/ml. These levels reflect those used in the original publication [7] and are equal to physiological levels in pregnant women. HCG concentrations in peripheral blood reach peak concentrations ~25,000–280,000 mIU hCG/ml (depending on the assay system) and gradually decline to ~3,000–20,000 mIU/ml for the remainder of pregnancy, [[7,8], product inserts]. Every 24 hrs, culture medium was changed and new medium containing hCG was added. HCG from Sigma was used in three experiments, from Calbiochem in six experiments, and from Cell Sciences Inc. in five experiments. To determine parasitaemia, either aliquots were collected every other day or sets of triplicate wells were harvested.

### Determining parasitaemia

In eight experiments, thin blood films were prepared, stained with Diff-Quick (IMEB Inc., San Marcos, CA). Percent parasitaemia was determined by two microscopists who examined coded smears and counted the number of IE per 500 to 1,000 RBC. In the remaining six experiments, cells were treated with 2 μl of 5 mM Vybrant DyeCycle™ Stain per 10^6 ^cells (Molecular Probes, Invitrogen) and the number of IE per 500,000 RBC was determined by flow cytometry using a FACSaria (Beckin-Deckinson).

### Co-culturing *P. falciparum *with BeWo cells

BeWo cells are a human choriocarcinoma cell line [13]. When treated with forskolin, they form syncytia with characteristics of syncytiotrophoblasts (BeWo-ST) and secrete hCG plus a variety of cytokines and hormones [13,15]. The BeWo-IE co-culture system was optimized prior to use. BeWo cells were cultured in HAM's F 12 complete medium supplemented with 2 mM L-glutamine, 100 units/mL of penicillin, 100 μg/mL of streptomycin, and 10% FBS. Prior to each experiment, 2.5 × 10^4 ^cells/ml were seeded in each well of a 48-well microtiter plate. After 24 h, the cells were induced with 40 μM of forskolin (Sigma, USA) for 48 hrs using FBS-free medium that was changed daily, and then cultured for 24 h in complete medium before the experiment. Microscopic examination of cell morphology and monitoring of hCG production were indicative of BeWo transformation into syncytia.

The parasitaemia was adjusted to the levels specified in the text. Then, an aliquot was either cultured in parasite culture medium as described above, or an equal volume of the IE was added to the monolayer of BeWo-ST cells in triplicate. Co-cultures were incubated at 37°C, with 5% CO_2_, 5% O_2_, and 90% N_2_. IE were harvested from the individual wells, stained with Vybrant DyeCycle™, and examined by flow cytometry.

### Measurement of hCG in co-cultures

The concentration of hCG in BeWo culture supernatants was determined using the Human Chorionic Gonadotropin-Beta Micro-ELISA Test kit (T108) and the accompanying hCG standards from Leinco Technologies (St. Louis, MO).

### SDS-PAGE analysis of commercial hCG

hCG from the three sources were analysed by SDS-PAGE according to Laemmli [16]. Ten μg of protein, based on protein concentration provided by the manufacturer, was added to reducing buffer, heated at 100°C for 5 minutes, and electrophoresed on pre-prepared Invitrogen Novex Bis-Tris 4–12% gels (Invitrogen, Carlsbad, CA). SeeBlue Plus2 pre-stained standards were used and gels were stained with Simply Blue Coomassie stain (both from Invitrogen).

### Data analysis

Percent parasitaemia, determined either by slide or FACS, was summarized by geometric means and geometric standard errors and pair-wise comparisons were performed between different concentrations of hCG and absence of hCG using Student's t test. The percent parasitaemia from IE, when cultured alone or with BeWo cells, was compared using analysis of variance (ANOVA) adjusted by Tukey's rule.

## Results

### SDS-PAGE analysis of commercial hCG

**Figure 1:**
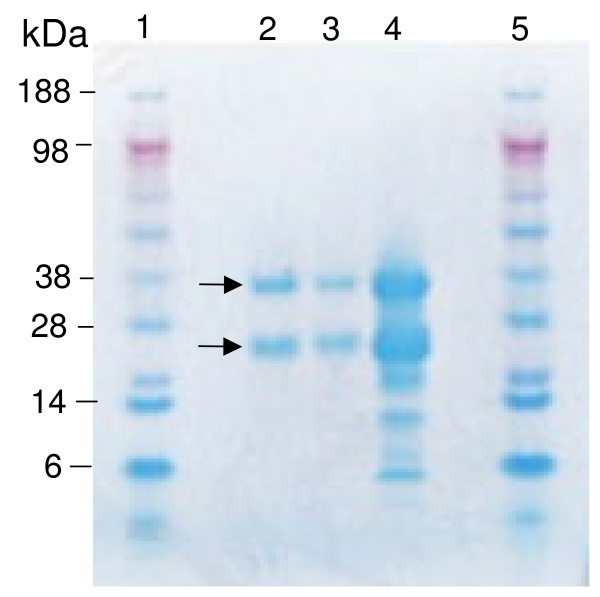
**SDS-PAGE of hCG used in the study**. The lanes contain: 1) molecular weight pre-stained standards, 2) hCG from Sigma Aldrich, 3) Calbiochem, 4) Cell Sciences, Inc., and 5) molecular weight standards. Arrows indicate location of the β- and α-chains of hCG.

The three commercial sources of hCG were compared by SDS-PAGE under reducing conditions. Since hCG is a glycoprotein with multiple glycosylation sites, including two in the alpha chain (MW 14.5 kDa) and four in the beta chain (MW 22.2)], the subunits do not run accurately on SDS-PAGE [17]. Results show that hCG from Sigma and Calbiochem consisted primarily of the alpha and beta-chains of hCG, whereas additional bands were present in the preparation from Cell Sciences, Inc. (Figure 1).

### Influence of hCG on in vitro growth of *P. falciparum*

**Figure 2:**
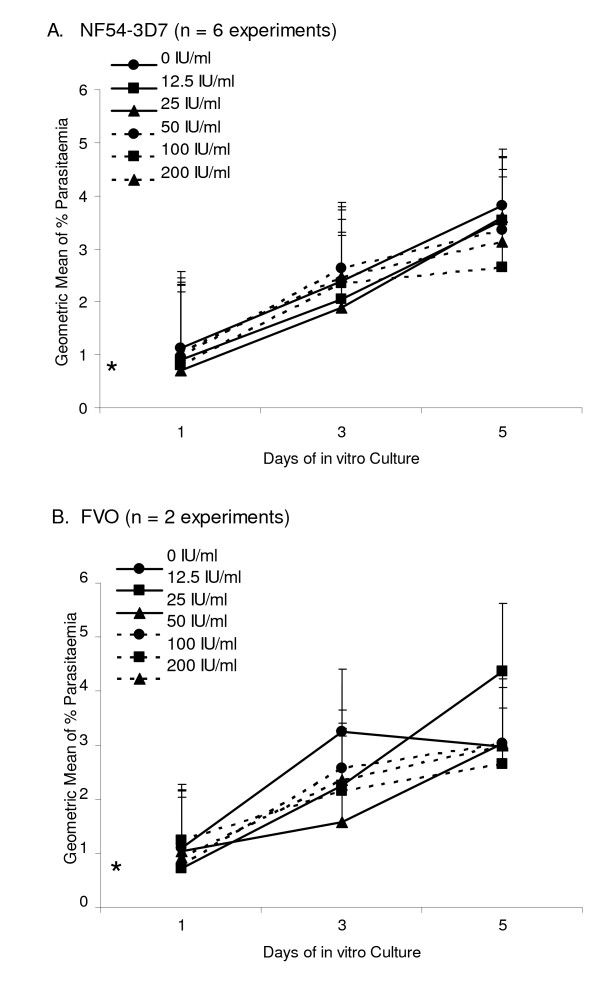
**Influence of hCG on parasite growth *in vitro *with parasitaemia determined by microscopy**. **A)**. Geometric mean parasitaemia ± SE for NF54-3D7 parasites cultured with hCG from Calbiochem (3 experiments), Cell Sciences, Inc. (2 exp't) and Sigma Aldrich (1 exp't); results from triplicate cultures were averaged and then the means for each of the 6 expt. were averaged. **B) **FVO parasites were cultured with hCG from Calbiochem (1 exp't) and Cell Sciences (1 exp't.); results from triplicate wells were averaged and then the means from each experiment were averaged. The * indicates starting parasitaemia.

In eight experiments, the 3D7 and FVO strains of *P. falciparum *were cultured with various concentrations of hCG and parasitaemia was assessed by microscopy. Comparison of the results reveals no difference between cultures supplemented with hCG from the three difference sources, so the results for each parasite strain were averaged. Overall, during the first three days of *in vitro *culture, average parasitaemia in cultures without hCG rose from 0.5% on Day 0 to 2.4% (3D7) and 3.2% (FVO) on Day 3, and then the growth of FVO slowed slightly (Figure 2A and 2B, respectively). No significant difference in parasite growth was found in cultures supplemented with increasing concentrations of hCG (12.5 – 200 IU/ml) compared to that in the absence of hCG on Day 1, 3, and 5, (all pair-wise p values were > 0.05 using Student's t-test). Therefore, the addition of 12.5 to 200 IU/ml of hCG to parasite cultures neither enhanced nor suppressed parasite growth *in vitro *based on microscopic assessment of parasitaemia.

**Figure 3:**
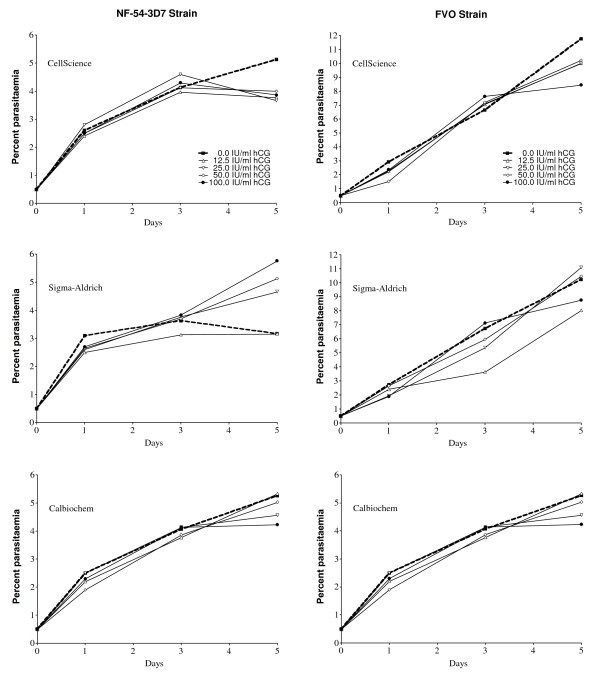
**Influence of hCG on parasite growth *in vitro *with parasitaemia determined by flow cytometry**. Geometric means for experiments in which 3D7 and FVO parasites were cultured with hCG from the three sources. Results from triplicate wells were averaged for cultures without hCG (dotted lines) and cultures supplemented with hCG (solid lines).

Since parasitaemia based on microscopy can produce variable results especially with low parasitaemia, the study was repeated using flow cytometry where the number of IE per 500,000 RBC was determined (Figure 3). In addition, in the same experiment cultures of NF54-3D7 and FVO were supplemented with hCG from CellSciences, Sigma-Aldrich, and Calbiochem (Figure 3). Starting parasitaemia of 0.5% increased to 4.1% for 3D7 and 10% for FVO during the period of culture. The higher parasitaemia in these cultures compared to those in Figure 2 reflects the improved accuracy of flow cytometry in estimating parasitaemia compared to microscopy. No difference was found in parasitaemia when NF54-3D7 and FVO (Figure 3) parasites were cultured with 12.5 to100 IU hCG/ml from each of the three sources (P > 0.05 in all pair-wise comparisons).

### Co-culture of *P. falciparum *with human chorionic BeWo cells

**Figure 4:**
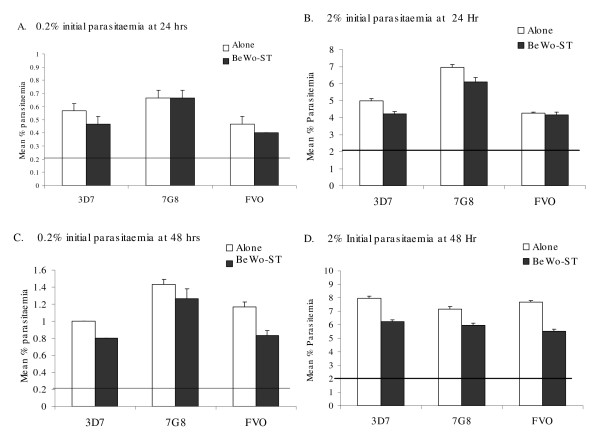
**Co-culture of BeWo cells with *P. falciparum*-infected erythrocytes**. Parasites were either cultured normally (alone) or with BeWo-ST. Parasitaemia after 24 hrs in cultures initiated with (A) 0.2% parasitaemia or (B) 2% parasitaemia. Parasitaemia after 48 hrs in cultures initiated with (C) 0.2% or (D) 2% parasitaemia. Results are means percent parasitaemia ± SD for triplicate samples determined by flow cytometry. Horizontal lines indicate initial parasitaemia.

Aliquots containing 0.2% and 2.0% parasitaemia of 3D7, 7G8, and FVO *P. falciparum*-IE were added to the syncytialized BeWo-ST cells, so that the IE made direct contact with cells secreting hCG. IE were also cultured without BeWo-ST cells. After 24 hours, co-cultures initiated with 0.2% and 2% parasitaemia contained 6,510 ± 643 and 5,875 ± 180 mIU hCG/ml, respectively; with similar levels detected at 48 hrs (6,100 ± 473 and 3,950 ± 512 mIU hCG/ml, respectively). The 3D7, 7H8 and FVO strains grew at different rates, with parasitaemia increasing 2- to 3.3-fold during the first 24 hrs in cultures with and without BeWo-ST cells (Figure 4A–B). Instead of enhancing parasite growth, the parasites grew at a slightly slower rate in co-cultures with hCG-secreting BeWo-ST cells. Parasitaemia continued to increase during the next 24 hrs in all cultures except for one (7G8 strain initiated at 2% parasitaemia), but parasitaemia were consistently lower in the co-cultures (Figure 4C and 4D) than in the cultures of IE is complete parasite culture medium. Thus, there was no evidence that parasites were stimulated to grow faster during a 48 hr period in this co-culture system.

## Discussion

A previous study reported that NF54 *P. falciparum *parasites grew faster *in vitro *when 8.3 and 16.7 IU hCG/ml from Ferring were added to the cultures [7]. The response to hCG was dose-dependent, eliminated by boiling, but suppressive above 33 IU/ml (or as reported, 200 IU/6 ml). In the current study, addition 12–200 IU/ml of commercial hCG from three sources did not alter the growth of the NF54-3D7 or FVO strains of *P. falciparum in vitro *(Figures 2 and 3). IE of the 3D7, 7G8, and FVO strains were also co-cultured with the BeWo-ST in an attempt to replicate conditions within the IVS where hCG is produced. Parasitaemia were actually lower, not enhanced, in the co-cultures compared to parasitaemia in routine cultures conducted at the same time (Figure 4). BeWo-ST secrete many bioactive factors in addition to hCG [15]. It is unclear if the lower parasitaemia in the co-cultured of BeWo and IE were due to factors produced by the BeWo cells or if culture conditions in the co-cultures were sub-optimal for extended parasite growth due to the addition of BeWo culture medium. Taken together, the results provide little evidence that hCG, either purified or naturally produced, enhances the rate of parasite growth.

Additional support for the conclusion that hCG does not enhance parasite growth comes from a search of the *P. falciparum *(3D7) genome database. If malarial parasites were able to respond to hCG, they should have a receptor for the hormone. A nucleotide BLAST, (Basic Local Alignment Search Tool) search found no similarities between the genome of *P. falciparum *and the luteinizing hormone/choriogonadotropin receptor (LHCGR) (NM 000233.3 – mRNA). Thus, this parasite does not have a receptor that shares homology with the human receptor for hCG. A careful search of literature also found no reports that hCG enhances growth of any other protozoan parasite.

It is difficult to determine why results of the current study failed to confirm those of the earlier one [7]. A diligent attempt was made to replicate the previous study. The most obvious difference was the source of hCG. The hCG preparations used in this study consisted primarily of the α and β-chains of hCG, although they could have been contaminated with other molecules (Figure 1). Previous studies have reported significant variation in purity and biological activity of commercial hCG preparations [11,12,17]. A comparative study examined hCG obtained from Ferring (Choragon) with other commercial sources and found that it was contaminated with a high level of EGF [10]. An other study reported hCG from Ferring stimulated CHO cells to produce cAMP, possibly due to the presence of the nicked form of hCG [18]. The preparations used in these studies and those of Rohrig *et al *[7] could be different, but it is possible that the effect on parasite growth they found was due to highly glycosylated or nicked-forms of hCG or to other contaminating stimulatory molecule in the preparation. Recombinant hCG has been used in clinical trials [19], but is not readily available for research purposes. Although the differences remain unclear, the three preparations of purified hCG tested in the current study did not enhance the growth of *P. falciparum in vitro*.

Even though hCG may not have a direct effect on IE, it could still be important in placental malaria. The primary role of hCG is to extend the life of the *corpus luteum*, most likely by increasing endothelial cell proliferation and vessel stabilization, and participate in early placental angiogenesis [20]. Thus, hCG may have a role in creating new blood vessels where trophozoite-stage *P. falciparum *IE sequester. HCG has also been found to modulate innate and acquired immune responses (reviewed in [21]). Accordingly, it is possible an increase in parasite numbers in the peripheral blood of pregnant women results from suppression of immune responses that aid in parasite clearance.

In summary, pregnant women are more susceptible to malaria and have higher parasitaemia than other adults. IE sequester in the IVS by adhesion to CSA and reach high numbers in the placenta, especially early in pregnancy before antibodies to var2csa are produced. At this time, ring-stage parasites are released from the IVS into the peripheral blood, thereby increasing peripheral parasitaemia. These events correspond with peak hCG levels. The cytoadherence mechanism along with a decrease in immune responses that control parasitaemia are sufficient to explain why pregnant women are susceptible to higher parasitaemia during the early part of pregnancy.

## Conclusion

Results from the study failed to conclusively demonstrate a role of hCG in enhancing the growth of *P. falciparum in vitro*. It is, therefore, unlikely that increased parasitaemia found early in pregnancy is due to hCG-mediated enhancement of parasite growth.

## Competing interests

The authors declare that they have no competing interests.

## Authors' contributions

CKP and ADT were responsible for study design, conducting the *in vitro *culture experiments, and helping in manuscript preparation; JAZ preformed statistical analysis of the data, and DWT assisted in study design, data interpretation and manuscript preparation.
